# Clinical significance of mechanistic target of rapamycin expression in vessels that encapsulate tumor cluster‐positive hepatocellular carcinoma patients who have undergone living donor liver transplantation

**DOI:** 10.1002/ags3.12735

**Published:** 2023-08-28

**Authors:** Katsuya Toshida, Shinji Itoh, Takeo Toshima, Shohei Yoshiya, Ryoichi Goto, Atsuyoshi Mita, Noboru Harada, Kenichi Kohashi, Yoshinao Oda, Tomoharu Yoshizumi

**Affiliations:** ^1^ Department of Surgery and Science, Graduate School of Medical Sciences Kyushu University Fukuoka Japan; ^2^ Department of Gastroenterological Surgery I Hokkaido University Graduate School of Medicine Sapporo Japan; ^3^ Division of Gastroenterological, Hepato‐Biliary‐Pancreatic, Transplantation, and Pediatric Surgery, Department of Surgery Shinshu University School of Medicine Nagano Japan; ^4^ Department of Anatomic Pathology, Graduate School of Medical Sciences Kyushu University Fukuoka Japan

**Keywords:** everolimus, hepatocellular carcinoma, living donor liver transplantation, mechanistic target of rapamycin, vessels that encapsulate tumor cluster

## Abstract

**Background:**

There is limited published information regarding the expression of mechanistic target of rapamycin (mTOR) in vessels that encapsulate tumor cluster (VETC)‐positive hepatocellular carcinoma (HCC). The mTOR inhibitor, everolimus, has been approved as an immunosuppressant for use in HCC patients after living donor liver transplantation (LDLT).

**Methods:**

Using a database of 214 patients who underwent LDLT for HCC, we examined the mTOR protein and angiopoietin‐2 (Ang‐2) in VETC‐positive HCC by immunohistochemical staining. The presence of VETC and mTOR expression were evaluated in both primary and recurrent HCC lesions.

**Results:**

Forty‐three of the 214 patients (20.1%) were VETC‐positive, and 29 of these 43 patients (67.4%) expressed mTOR. Relative Ang‐2 expression was significantly higher in the mTOR‐positive than in the mTOR‐negative group (*p* = 0.037). Thirty‐four of the 214 patients experienced HCC recurrence after LDLT; 20 of these were operable. The primary lesions of six of these 20 patients were VETC‐positive; five of these six patients also had VETC‐positive recurrent lesions (*p* < 0.001). The expression of mTOR was significantly higher in the VETC‐positive lesions (*p* = 0.0018).

**Conclusions:**

We showed that mTOR expression was higher in the VETC‐positive primary and recurrent lesions than in the VETC‐negative ones.

## Graphical abstract

mTOR expression is high in patients with hepatocellular carcinoma (HCC) that have vessels that encapsulate tumor clusters, suggesting that the mTOR inhibitor, everolimus, could be particularly affective in patients with HCC being treated using living donor liver transplantation.

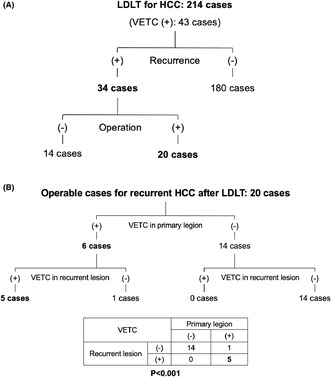

## 1 INTRODUCTION

Hepatocellular carcinoma (HCC) is a primary liver malignancy and the third most common cause of cancer‐related mortality.^1^ Hepatic resection is performed as a curative treatment for HCC, but liver transplantation (LT) has been accepted as the effective treatment strategy with strict adherence to the widely used Milan criteria.^2^, ^3^, ^4^ Despite the high incidence of HCC recurrence within the LT population, there is no established evidence to guide specific post‐LT surveillance strategies. Almost all LT patients require immunosuppressive medication, which lowers their anti‐tumor immunity and frequently leads to intrahepatic or extrahepatic HCC recurrence.^5^

The existence of vessels that encapsulate tumor clusters (VETC) was first reported by Fang et al. and VETC presence is associated with rapid tumor dissemination and high recurrence rates in HCC.^6^ Some studies have highlighted the relationship between VETC and the tumor immune microenvironment.^7^, ^8^ In a study of living donor LT (LDLT) for HCC, Toshima et al. showed the prognostic impact of VETC on the frequencies of tumor‐infiltrating lymphocytes. Thus, VETC could represent a novel prognostic biomarker to predict the outcome of HCC patients after LDLT.^9^

The mechanistic target of rapamycin (mTOR) has various roles in biological processes such as cell proliferation, survival, autophagy, metabolism, and immunity. Thus, the mTOR signaling pathway is a promising target in anti‐tumor therapy.^10^ Because mTOR inhibitors differ in their mechanism of action from calcineurin inhibitors and exhibit potent immunosuppressive effects, they have increasingly been used in clinical practice in the context of LDLT.^11^

To the best of our knowledge, there is limited published information on the association between VETC and mTOR expression in HCC patients treated with LDLT or the roles of VETC and mTOR in recurrent lesions. The aim of the present study was therefore to address these questions and examine whether the use of mTOR inhibitors as immunosuppressive drugs could prevent or reduce the recurrence of HCC after LDLT.

## 2 MATERIALS AND METHODS

### 2.1 Patients and specimen preparation

This retrospective study was approved by the Ethics Committee of Kyushu University Hospital. Two hundred and fourteen patients, who had undergone LDLT for HCC at Kyushu University Hospital (Fukuoka, Japan), Shinshu University Hospital (Nagano, Japan), and Hokkaido University Hospital (Sapporo, Japan) between January 1999 and December 2021, were retrospectively selected for this study. The selection criteria for LDLT in HCC patients were as follows: (1) LDLT was the only available treatment option for HCC patients; (2) absence of extrahepatic metastasis; and (3) absence of major vascular infiltrations. In accordance with the Kyushu University criteria, LDLT was not performed in HCC patients with tumor sizes >5 cm and des‐γ‐carboxy prothrombin (DCP) levels >300 mAU/mL.^12^ The detailed surgical procedure and pro/peri/postoperative management for LDLT have been previously described.^13^, ^14^ The specimens were fixed in 10% formalin solution, embedded in paraffin, and sectioned into 4‐μm‐thick slices to evaluate the histological features.

### 2.2 Collection of clinicopathological characteristics

Patient clinical data such as age, sex, hepatitis B surface‐antigen (HBV‐Ag) status, hepatitis C virus‐antibody (HCV‐Ab) status, alcohol consumption, Model for End‐Stage Liver Disease (MELD) score, splenectomy, maximum tumor size, number of tumors, tumor distribution, α‐fetoprotein (AFP) levels, DCP levels, pre‐treatment, microvascular invasion, and poor differentiation were recorded.

### 2.3 Follow‐up strategy

After discharge, all patients underwent abdominal computerized tomography (CT) scanning every 3 months, and chest CT scanning, magnetic resonance imaging, positron emission tomography, and bone scintigraphy every 6 months for 5 years after LDTL or as needed. Overall survival (OS) was defined as death from any cause. Disease‐free survival (DFS) was defined as the time to HCC relapse after LDLT.

### 2.4 Immunohistochemistry (IHC)

IHC staining was performed on 4‐μm formalin‐fixed, paraffin‐embedded sections. The sliced sections were deparaffinized in xylene and rehydrated using a graded ethanol series. The specimens were subjected to antigen retrieval by heating to 121°C (in a microwave) for 15 min, in 10 mM citrate buffer (pH 6.0), followed by blocking with 10% normal goat serum. The tissue sections were then incubated at 4°C overnight with the following antibodies: mouse monoclonal anti‐CD34 (dilution 1:100, QBEnd/10, Santa Cruz Biotechnology) Biotechnology, Dallas, Texas, USA), mouse monoclonal anti‐mTOR antibody (dilution 1:2000, 66 888‐1‐Ig, Proteintech), and rabbit monoclonal anti‐angiopoietin‐2 (Ang‐2) (dilution 1:200, ab155106, Abcam). Color development was performed using 3,3‐diamino‐benzidine, followed by counterstaining with Mayer's hematoxylin. Slides were scanned using the NanoZoomer slide scanner (Hamamatsu Photonics KK). The VETC pattern was identified by the presence of vessels that formed cobweb‐like networks and that encapsulated individual tumor clusters.^15^ The five most intensely vascularized fields were screened using light microscopy (100× magnification) to evaluate the presence of a VETC pattern. Samples with a VETC pattern were labeled VETC(+) and those without a VETC pattern were labeled VETC(−). mTOR expression was graded according to anti‐mTOR antibody staining intensity: negative, weakly positive, moderately positive, or strongly positive; negative and weakly positive staining was classed as mTOR‐negative, whereas moderately positive and strongly positive staining was classed as mTOR‐positive. Ang‐2 expression was quantified using Image J software (National Institutes of Health, USA), as previously described. The density of Ang‐2 staining was assessed by quantifying the positive area of staining within the total surface area of the region of interest.^9^ We evaluated the staining of mTOR and Ang‐2 in the VETC(+) areas. In the case of VETC(−), evaluation was performed at the site of the strongest expression of mTOR/Ang2 in the tumor.

### 2.5 Western blotting (WB)

WB was performed as previously described.^16^ Briefly, total protein was extracted from surgically excised tumor specimens and denatured at 95°C for 5 min. The proteins were then separated by electrophoresis using SuperSep Ace 10% gels (Fujifilm) at 20 mA for 80 min. Next, the proteins were transferred onto a polyvinylidene difluoride membrane using the Trans‐Blot Turbo Transfer System (Bio‐Rad, Hercules, CA). Primary and secondary antibodies were diluted in iBind solution (Invitrogen) and applied to the membrane. The following primary antibodies were used: anti‐rabbit mTOR (dilution 1:1000, #2983, Cell Signaling Technology), anti‐rabbit phospho‐mTOR (dilution 1:1000, #5536, Cell Signaling Technology), and anti‐rabbit βactin (dilution 1:5000, GeneTex). The secondary antibody was goat anti‐rabbit IgG (H & L;dilution 1:5000; Abcam). Blots were developed using the Chemiluminescent HRP Antibody Detection Reagent (Denville Scientific) and imaged using the Amersham Imager 600 (GE Healthcare).

### 2.6 Statistical analysis

All statistical analyses were performed using SAS software (JMP Pro 16; SAS Institute Inc.). Categorical variables were reported as percentages and compared using Fisher's exact test. Cumulative DFS and OS rates were calculated using the Kaplan–Meier method, and differences between the curves were evaluated using the log‐rank test. *p*‐values <0.05 were considered as a measure of statistical significance.

## 3 RESULTS

### 3.1 Clinicopathological characteristics of patients with VETC‐positive HCC

**FIGURE 1:**
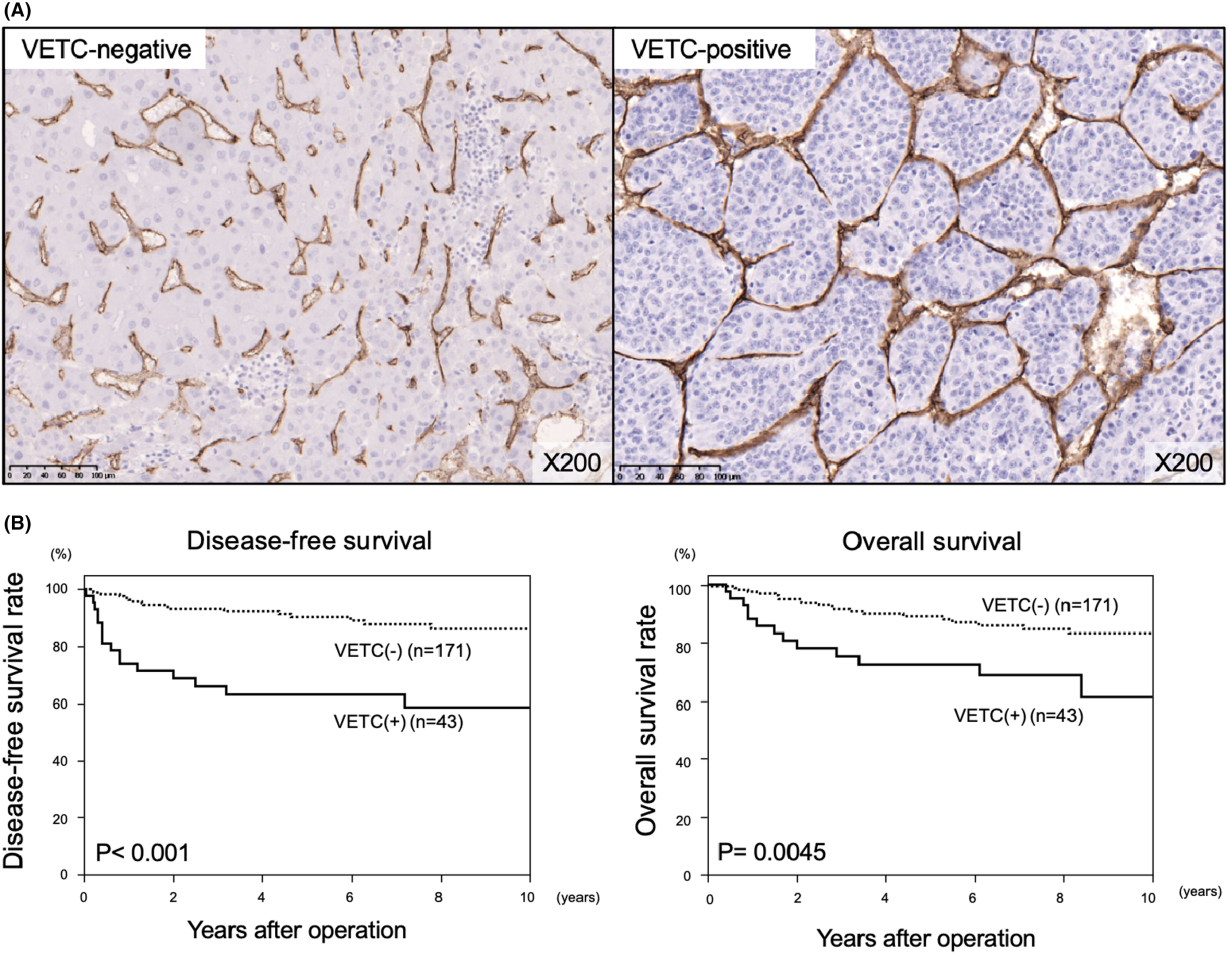
(A) Representative features of the vessels that encapsulate tumor clusters (VETC), demonstrated by immunohistochemical staining. VETC‐negative (left), VETC‐positive (right). (B) Kaplan–Meier analysis revealed that VETC‐positive HCC patients had significantly lower rates of disease‐free survival (DFS; *p* < 0.001) and overall survival (OS; *p* = 0.0045) than the VETC‐negative group. DFS, disease‐free survival; HCC, Hepatocellular carcinoma; OS, overall survival; VETC, vessels that encapsulate tumor clusters.

Figure 1A shows representative IHC staining of HCC tissues for VETC. Forty‐three of the 214 HCC patients were VETC(+). Kaplan–Meier analysis revealed that DFS (*p* < 0.001) and OS (*p* = 0.0045) were significantly lower in the VETC(+) group than in the VETC(−) group (Figure 1B). Univariate and multivariate analyses of the differences between the clinicopathologic factors and prognostic impact of the VETC(+) and VETC(−) groups have been reported previously, although the number of patients evaluated in previous study was smaller than in present study.^9^ In our study, of the 43 patients with HCC, 25 patients were male (58.1%) and the median age of the patients was 59 years (range 42–71 years). Other variables related to the recipients, donors, and tumors are shown in Table 1.

**TABLE 1:** Clinicopathological characteristics of patients with VETC‐positive HCC.

Variables	VETC‐positive (*n* = 43)
Recipient variables	
Age (years)	59 (42–71)
Sex, male/female	25/18
Etiology of liver failure	
HBs‐Ag positive	7 (16.3%)
HCV‐Ab positive	29 (67.4%)
ETOH	1 (2.3%)
Others	6 (14.0%)
MELD score	13 (12–32)
Splenectomy	34 (79.1%)
Donor variables	
Age (years)	36 (21–63)
Sex, male/female	24/19
Blood type: ABO incompatible (%)	4 (9.3%)
Graft: right lobe (%)	20 (46.5%)
GV/LV	39.9 (27.2–60.2)
Tumor variables	
Maximum tumor size (cm)	2.8 (1.0–6.6)
Number of tumors	3 (1–300)
Tumor distribution: bilobar (%)	24 (55.8%)
AFP (ng/mL)	201 (1.1–75 277)
DCP (mAU/mL)	98 (6–9693)
Pre‐treatment (%)	23 (53.5%)
Microvascular invasion (%)	24 (55.8%)
Poor differentiation (%)	27 (62.8%)

*Note*: Data are presented as *n* (%) or the median (range).

Abbreviations: AFP, α‐fetoprotein; DCP, des‐γ‐carboxy prothrombin; GV/LV, graft volume/liver volume; HBs‐Ag, hepatitis B surface antigen; HCV‐Ab, hepatitis C virus antibody; LDLT, living donor liver transplantation; MELD, Model for End‐Stage Liver Disease.

### 3.2 Expression of mTOR protein in VETC‐positive HCC

**FIGURE 2:**
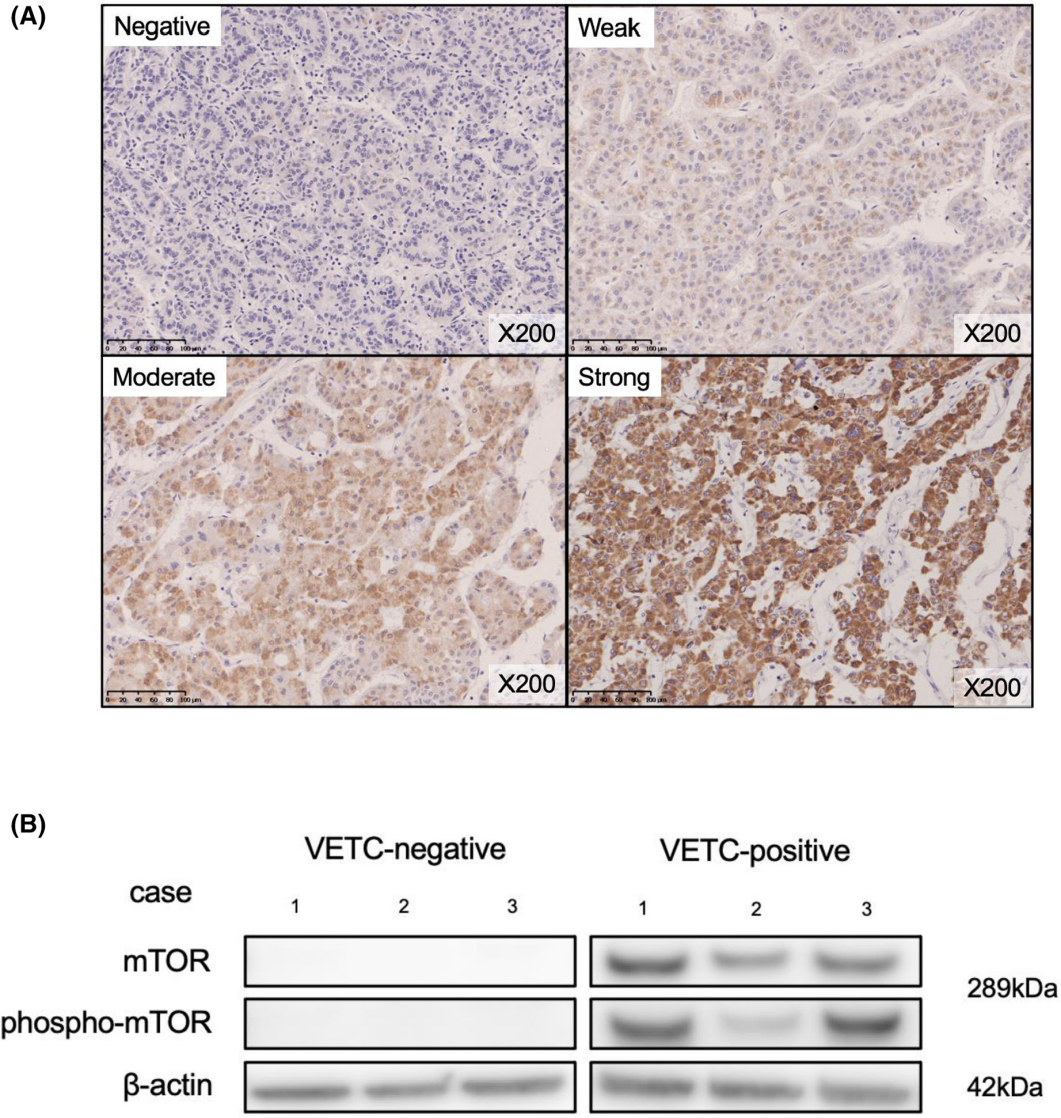
(A) Immunohistochemical staining of mTOR: negative staining/weak staining/moderate staining/strong staining (200× magnification). (B) Assessment of mTOR and phospho‐mTOR protein expression by western blotting. mTOR, mechanistic target of rapamycin.

We examined the expression of mTOR protein using IHC. The cytoplasmic expression of mTOR was evaluated in the VETC(+) areas of the tumor (Figure 2A). Of the 43 VETC(+) patients, 28 cases (65.1%) were mTOR‐positive, and of the 171 VETC(−) patients, 65 cases (38.0%) were mTOR‐positive, the mTOR positive rate was significantly higher in VETC (+) cases (*p* = 0.0013). Phosphorylation of mTOR was examined by WB. Three patients were randomly selected from each of the mTOR‐negative and ‐positive groups (*n* = 6). Proteins were then extracted from frozen tumor specimens to evaluate the expression mTOR and phospho‐mTOR in each sample. The three patients in the mTOR‐positive group were positive for mTOR and phospho‐mTOR expression, compared with the patients in the mTOR‐negative group, who did not express mTOR in either form (Figure 2B).

### 3.3 Correlation between mTOR and Ang‐2 levels

We next examined the correlation between mTOR and Ang‐2 levels in the VETC(+) HCC samples. The results of IHC staining for Ang‐2 were similar to those previously reported by Toshima et al.^9^ The relative Ang‐2 level was 0.13 (range: 0.09–0.19) in the mTOR‐negative group and 0.19 (range: 0.03–0.44) in the mTOR‐positive group, meaning that there was a significant difference between the two groups (*p* = 0.037, Figure S1). Similar to VETC(+) cases, the correlation between mTOR and Ang2 expression was examined in VETC(−) cases, no significant positive correlation was found between them.

### 3.4 The association between VETC and primary or recurrent HCC lesions

**FIGURE 3:**
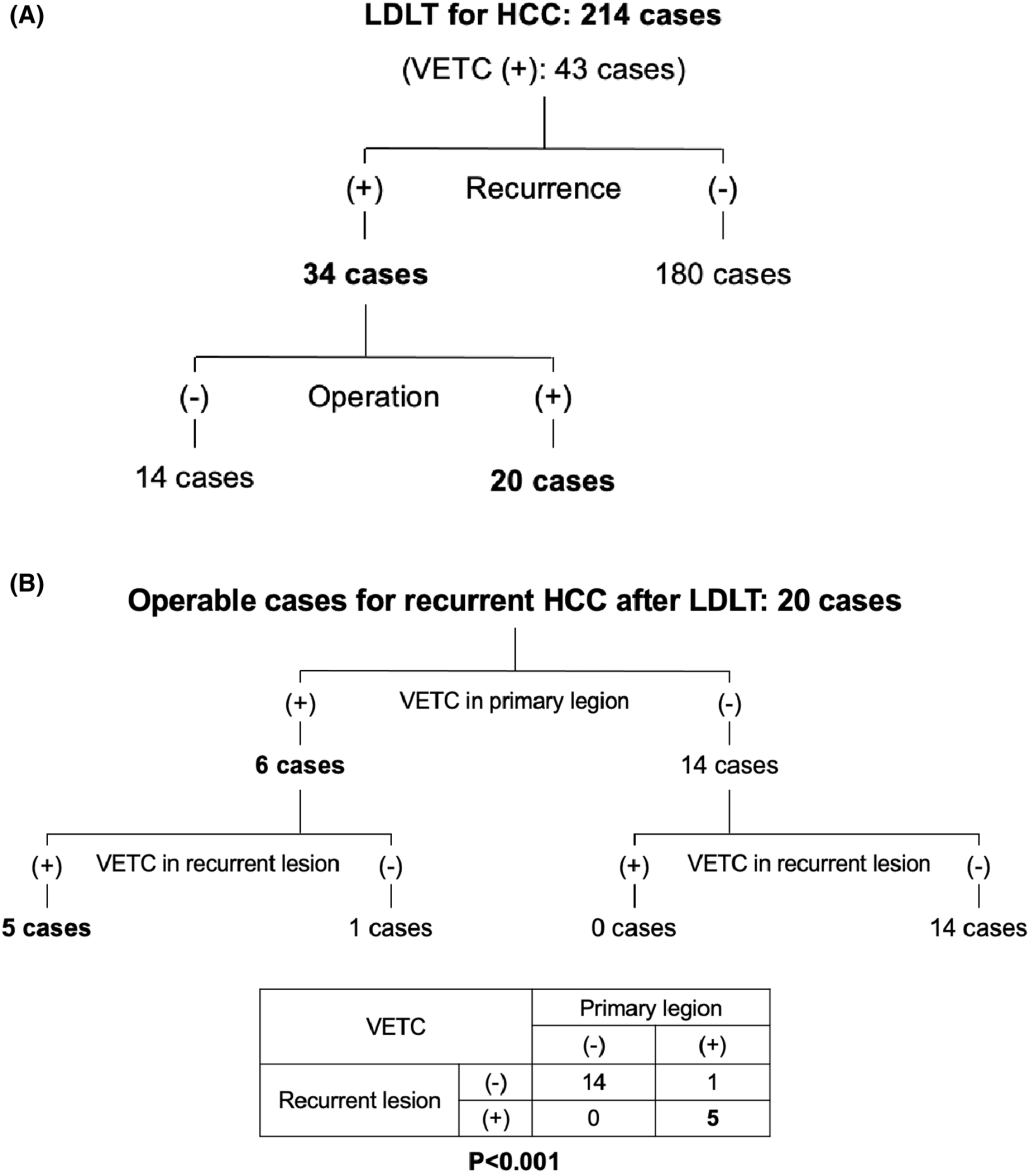
(A) Of the 214 patients who underwent LDLT for HCC, 34 experienced tumor recurrence; 20 of these 34 were operable. (B) Of the 20 patients who underwent surgery for recurrent lesions, three had VETC‐positive primary lesions. Conversely, patients who had VETC‐negative primary lesions were also had VETC‐negative recurrent lesions. HCC, Hepatocellular carcinoma; LDLT, living‐donor liver transplantation; VETC, vessels that encapsulate tumor clusters.

In this study, recurrent HCC after LDLT was observed in 34 out of the 214 patients. Of these 34 patients, 14 could not be operated on because of systemic metastases or poor liver function; however, 20 of the 34 patients underwent surgery (Figure 3A). The patients who could undergo surgery for recurrent lesions had significantly better OS rates (*p* < 0.001), measured from the time of detection of tumor recurrence after LDLT (Figure S2A). Six out of the 20 operable cases had VETC(+) primary lesions; five of these also had VETC(+) recurrent lesions. Notably, all the patients who had VETC(−) primary lesions also had VETC(−) recurrent lesions (Figure 3B). This result indicates that development of VETC in recurrent lesions is significantly associated with the presence of VETC in the primary lesions (*p* < 0.001). We investigated whether the presence or absence of VETC in recurrent lesions affected prognosis, and no significant difference was observed between the two groups (Figure S2B).

On the other hand, in the 14 patients who could not undergo surgery for recurrent lesions, 10 patients had VETC(+) primary lesions, and the presence of VETC did not significantly differ in the prognosis of them (Figure S2C).

### 3.5 The expression of mTOR in recurrent lesions

We next examined the expression of mTOR in recurrent lesions of the 20 operable HCC patients using IHC. We found that six of the 20 cases were mTOR‐positive, and that five of six mTOR‐positive cases had VETC(+) recurrent lesions, whereas one mTOR‐positive patient had VET(−) recurrent lesions. This indicate that VETC(+) recurrent lesions were significantly associated with a higher expression of mTOR (*p* = 0.0018). The information relating to the expression of mTOR in VETC+/− primary and recurrent lesions, and use of everolimus, is summarized in Table 2. None of the patients who did not undergo surgery for recurrent disease used everolimus. The patient (case 9 in Table 2) was started to be administered with eveololimus after radical surgery for the second recurrence of HCC. The third recurrence of HCC was observed, but the surgery was performed again, and no further recurrence was observed. The patient (case 10 in Table 2) was started to be administered with everolimus after radical resection of the first recurrent of HCC. She currently is undergoing systemic therapy for the second recurrent of HCC. Two patients, one VETC(+) and one VETC(−), are doing well, although with recurrence after introduction of everolimus.”

**TABLE 2:** Clinicopathological characteristics of patients who underwent operation for recurrent HCC after LDLT.

Case	Age	Sex	Splenectomy (at LDLT)	Differentiation (primary)	Recurrent lesion	Use of Everolimus	VETC (primary)	mTOR (primary)	VETC (recurrent)	mTOR (recurrent)
1	58	F	No	Moderate	Liver	No	−	−	−	+
2	59	F	No	Moderate	Liver	No	−	−	−	−
3	58	F	No	Well	Rectum, Lymph node	No	−	−	−	−
4	51	M	Yes	Poor	Lung	No	+	+	−	−
5	65	M	Yes	Poor	Rectum	No	−	−	−	−
6	47	F	Yes	Poor	Ileum	No	−	−	−	−
7	59	F	No	Poor	Lung	No	−	−	−	−
8	64	F	Yes	Poor	Lung	No	+	+	+	+
9	47	F	Yes	Moderate	Liver	No	+	−	+	+
10	48	M	Yes	Moderate	Lung	Yes	+	+	+	+
11	64	F	Yes	Poor	Lung	Yes	−	−	−	−
12	54	M	Yes	Poor	Liver	No	−	−	−	−
13	63	M	Yes	Poor	Liver, Lymph node	No	−	−	−	−
14	64	M	No	Moderate	Adrenal gland	No	−	+	−	−
15	52	F	No	Moderate	Lung	No	−	−	−	+
16	47	M	No	Moderate	Lung	No	−	−	−	−
17	40	M	No	Poor	Adrenal gland	No	−	−	−	−
18	55	M	No	Poor	Lymph node	No	+	+	+	+
19	62	M	No	Poor	Lung	No	−	−	−	−
20	62	M	Yes	Poor	Lung	No	+	+	+	+

Abbreviations: HCC, hepatocellular carcinoma; LDLT, living donor liver transplantation; mTOR, mechanistic target of rapamycin; VETC, vessels that encapsulate tumor cluster; Primary, primary lesion, Recurrent, Recurrent lesion.

## 4 DISCUSSION

In the present study, we immunohistologically examined the expression of mTOR in HCC patients with VETC(+) primary and recurrent lesions after LDLT. We demonstrated that mTOR was expressed in 65.1% of the VETC(+) HCC patients and that mTOR expression was significantly associated with the development of VETC in recurrent lesions.

The concept of VETC was first reported by Fang et al.^6^ VETC is present in 16.9%–23.8% of HCC patients and is a poor prognostic factor in HCC.^17^, ^18^, ^19^ In the present study, 20.1% of HCC patients who underwent LDLT for HCC had VETC(+) tumors; this figure is in agreement with previous reports. VETC(+) HCC is highly malignant and its occurrence is correlated with programmed cell death ligand 1 (PD‐L1) and vascular endothelial growth factor expression. Therefore, there is an urgent need to develop effective treatment options for VETC(+) HCC patients.^7^, ^8^ Epithelial‐mesenchymal transition signatures are rarely observed in VETC(+) cases. In addition, although VETC has been shown to provide an efficient metastatic mode by facilitating the release of the entire tumor mass into the bloodstream, it is unclear whether metastatic sites exhibit similar morphology. Moreover, there are no reports documenting the role of VETC in HCC recurrent lesions and its involvement in primary lesions. Although our findings were based on a small sample size, we showed that HCC patients with VETC(+) primary lesions were more likely to develop VETC(+) recurrent lesions. However, one limitation of our study was that the tumors evaluated belonged to HCC patients in which surgery was feasible; it is possible that the tumors of inoperable patients were more malignant and had a higher incidence of VETC. The development of imaging or blood testing methods to identify VETC(+) tumors would enable the more detailed study of these tumors for the potential benefit of HCC patients.

To date, LDLT has been performed as an effective treatment for HCC. The use of immunosuppressive medications is essential after LT to prevent organ rejection. However, this often leads to HCC relapse due to decreased antitumor immunity.^20^ In fact, the use of calcineurin inhibitors as immunosuppressants has been reported to increase the recurrence of HCC.^21^, ^22^ In such instances, a 3‐month treatment course with an mTOR inhibitor has been shown to improve the outcome of LT for HCC, especially in patients with evidence of high AFP activity within the tumor.^23^ Moreover Jeng et al. reported that the introduction of everolimus accompanied by a reduction in tacrolimus dose was non‐inferior to standard tacrolimus treatment in terms of efficacy and renal function at 12 months. Moreover, using this treatment regimen, HCC recurrence was observed only in tacrolimus control patients.^24^ mTOR is a protein kinase, which is involved in the regulation of cell growth, survival, metabolism, and immunity. Therefore, the uncontrolled activation of mTOR promotes tumor growth and metastasis.^25^ Studies have shown that the mTOR inhibitor, everolimus, inhibits the growth, migration, and invasiveness of HCC cell lines.^26^, ^27^ Moreover, HCC‐cell‐derived Ang‐2 is a prerequisite for VETC formation. Knockdown of Ang‐2 using microRNA‐100 was shown to reduce Ang‐2 protein levels by targeting mTOR, which in turn inhibited the formation of VETC.^28^ Thus, inhibition of mTOR not only plays an important role in the control of cancer progression in HCC but also is closely associated with the formation of VETC. In the present study, mTOR was positive in 65.9% of patients who underwent LDLT for HCC in the group, and Ang‐2 levels were significantly higher in the mTOR‐positive group than mTOR‐negative group. The expression of mTOR was also significantly higher in the VETC‐positive group when examining recurrence lesions. Based on the above, the use of Everolimus after LDLT may prevent recurrence and exert an antitumor effect after recurrence in patients with VETC‐positive HCC. Recently, the combination of immune check inhibitor (ICI) atezolizumab (ATZ) and bevacizumab (BEV) has been used for unresectable HCC.^29^ Although ICI plays a role in activating the actions of tumor infiltrating lymphocytes (TILs), there is no report that ATZ/BEV was used for treatment for recurrent HCC after LDLT because it enhances the risk of postoperative rejection. On the other hand, Murakami et al. reported that the using mTOR inhibitor in ICI treatment can suppress rejection after renal transplantation.^30^ In the future, in the treatment of HCC recurrence after LT, the combination of ICI and everolimus may be important in suppressing rejection and maintaining function of TILs. Since the use of mTOR after LDLT began in Japan in 2018, real clinical studies on the inhibitory and therapeutic effects of everolimus on HCC after LDLT are scarce. Further study is desirable based on the accumulation of more cases in the future. Thus, the inhibition of mTOR not only plays an important role in the control of HCC progression but is also closely associated with the formation of VETC. In the present study, mTOR was expressed in 65.9% of the HCC patients who underwent LDLT. In addition, the relative Ang‐2 levels were significantly higher in the mTOR‐positive group than in mTOR‐negative group. The expression of mTOR was also significantly higher in the VETC(+) group of HCC patients with recurrent lesions. Based on the above findings, the use of everolimus after LDLT may prevent or reduce tumor recurrence in patients with VETC(+) HCC. Although the use of mTOR inhibitors in HCC patients treated with LDLT was approved in Japan in 2018, clinical studies documenting the inhibitory and therapeutic effects of everolimus in this context remain scarce. Further research is therefore needed as more HCC patients undergo this treatment in the future.

In conclusion, in this study we showed that mTOR expression was high in VETC (+) primary and recurrent HCC lesions, which has implications for the future treatment of HCC patients.

## AUTHOR CONTRIBUTIONS

K.T. participated in the study conception and design, analysis, and drafting of the article. S.I. participated in the study conception and design, and in the critical revision of the manuscript. T.T., S.Y., R.G., A.M., N.H., and K.K. participated in the data acquisition, analysis, and interpretation. Y.O and T.Y. participated in the critical revision of the manuscript.

## FUNDING INFORMATION

This study was supported by JSPS KAKENHI grant number JP‐19 K09198 and 22 K15544. The funding sources had no role in the collection, analysis, or interpretation of the data or in the decision to submit the article for publication.

## CONFLICT OF INTEREST STATEMENT

K.T. and other co‐authors have no conflict of interest.

## ETHICS APPROVAL AND INFORMED CONSENT

This retrospective study was approved by the ethics committee of Kyushu University (approval code: 23076–00).

## Supporting information


Appendix S1.

## References

[1] McGlynn KA , Petrick JL , El‐Serag HB . Epidemiology of hepatocellular carcinoma. Hepatology. 2021;73(S1):4–13. 10.1002/hep.31288 PMC7577946 32319693

[2] Taketomi A , Soejima Y , Yoshizumi T , Uchiyama H , Yamashita YI , Maehara Y . Liver transplantation for hepatocellular carcinoma. J Hep Bil Pancr Surg. 2008;15(2):124–130. 10.1007/s00534-007-1296-4 18392704

[3] Yonemura Y , Yoshizumi T , Inokuchi S , Kosai‐Fujimoto Y , Harada N , Itoh S , et al. Predictor of outcome after living donor liver transplantation for patients with hepatocellular carcinoma beyond the Japan criteria. Ann Gastroenterological Surg. 2020;4(4):413–421. 10.1002/ags3.12335 PMC7382431 32724885

[4] Yoshizumi T , Itoh S , Shimokawa M , Inokuchi S , Harada N , Takeishi K , et al. Simultaneous splenectomy improves outcomes after adult living donor liver transplantation. J Hepatol. 2021;74(2):372–379. 32827564 10.1016/j.jhep.2020.08.017

[5] Hoffman D , Mehta N . Recurrence of hepatocellular carcinoma following liver transplantation. Expert Rev Gastroent. 2021;15(1):91–102. 10.1080/17474124.2021.1823213 32933351

[6] Fang J , Zhou H , Zhang C , Shang L , Zhang L , Xu J , et al. A novel vascular pattern promotes metastasis of hepatocellular carcinoma in an epithelial–mesenchymal transition–independent manner. Hepatology. 2015;62(2):452–465. 25711742 10.1002/hep.27760

[7] Itoh S , Yoshizumi T , Yugawa K , Imai D , Yoshiya S , Takeishi K , et al. Impact of immune response on outcomes in hepatocellular carcinoma: association with vascular formation. Hepatology. 2020;72(6):1987–1999. 32112577 10.1002/hep.31206

[8] Kurebayashi Y , Matsuda K , Ueno A , Tsujikawa H , Yamazaki K , Masugi Y , et al. Immunovascular classification of HCC reflects reciprocal interaction between immune and angiogenic tumor microenvironments. Hepatology. 2022;75(5):1139–1153. 34657298 10.1002/hep.32201

[9] Kawasaki J , Toshima T , Yoshizumi T , Itoh S , Mano Y , Wang H , et al. Prognostic impact of vessels that encapsulate tumor cluster (VETC) in patients who underwent liver transplantation for hepatocellular carcinoma. Ann Surg Oncol. 2021;28(13):8186–8195. 34091774 10.1245/s10434-021-10209-5

[10] Saxton RA , Sabatini DM . mTOR signaling in growth, metabolism, and disease. Cell. 2017;168(6):960–976. 28283069 10.1016/j.cell.2017.02.004 PMC5394987

[11] Parlakpinar H , Gunata M . Transplantation and immunosuppression: a review of novel transplant‐related immunosuppressant drugs. Immunopharm Immunot. 2021;43(6):651–665. 10.1080/08923973.2021.1966033 34415233

[12] Taketomi A , Sanefuji K , Soejima Y , Yoshizumi T , Uhciyama H , Ikegami T , et al. Impact of des‐gamma‐Carboxy prothrombin and tumor size on the recurrence of hepatocellular carcinoma after living donor liver transplantation. Transplantation. 2009;87(4):531–537. 19307789 10.1097/TP.0b013e3181943bee

[13] Toshima T , Taketomi A , Ikegami T , Fukuhara T , Kayashima H , Yoshizumi T , et al. V5‐drainage‐preserved right lobe grafts improve graft congestion for living donor liver transplantation. Transplant J. 2012;93(9):929–935. 10.1097/TP.0b013e3182488bd8 22461038

[14] Imai D , Yoshizumi T , Sakata K , Ikegami T , Itoh S , Harada N , et al. Long‐term outcomes and risk factors after adult living donor liver transplantation. Transplantation. 2018;102(9):e382–e391. 29912047 10.1097/TP.0000000000002324

[15] Fang J , Xu L , Shang L , Pan C , Ding J , Tang Y , et al. Vessels that encapsulate tumor clusters (VETC) pattern is a predictor of Sorafenib benefit in patients with hepatocellular carcinoma. Hepatology. 2019;70(3):824–839. 30506570 10.1002/hep.30366

[16] Itoh S , Maeda T , Shimada M , Shin‐ichi A , Shirabe K , Tanaka S , et al. Role of expression of focal adhesion kinase in progression of hepatocellular carcinoma. Clin Cancer Res. 2004;10(8):2812–2817. 15102689 10.1158/1078-0432.ccr-1046-03

[17] Renne SL , Woo HY , Allegra S , Rudini N , Yano H , Donadon M , et al. Vessels encapsulating tumor clusters (VETC) is a powerful predictor of aggressive hepatocellular carcinoma. Hepatology. 2020;71(1):183–195. 31206715 10.1002/hep.30814

[18] Itoh S , Yoshizumi T , Kitamura Y , Yugawa K , Iseda N , Shimagaki T , et al. Impact of metabolic activity in hepatocellular carcinoma: association with immune status and vascular formation. Hepatology Commun. 2021;5(7):1278–1289. 10.1002/hep4.1715 PMC8279470 34278175

[19] Guan R , Lin W , Zou J , Mei J , Wen Y , Lu L , et al. Development and validation of a novel nomogram for predicting vessels that encapsulate tumor cluster in hepatocellular carcinoma. Cancer Control J Moffitt Cancer Cent. 2022;29:10732748221102820. 10.1177/10732748221102820 PMC9136459 35609265

[20] Yoshizumi T , Harimoto N , Itoh S , Okabe H , Kimura K , Uchiyama H , et al. Living donor liver transplantation for hepatocellular carcinoma within Milan criteria in the present era. Anticancer Res. 2016;36(1):439–445. 26722079

[21] Vivarelli M , Cucchetti A , Barba GL , Ravaioli M , Gaudio MD , Lauro A , et al. Liver transplantation for hepatocellular carcinoma under Calcineurin inhibitors. Ann Surg. 2008;248(5):857–862. 18948815 10.1097/SLA.0b013e3181896278

[22] Vivarelli M , Dazzi A , Zanello M , Cucchetti A , Cescon M , Ravaioli M , et al. Effect of different immunosuppressive schedules on recurrence‐free survival after liver transplantation for hepatocellular carcinoma. Transplantation. 2010;89(2):227–231. 20098287 10.1097/TP.0b013e3181c3c540

[23] Schnitzbauer AA , Filmann N , Adam R , Bachellier P , Bechstein WO , Becker T , et al. mTOR inhibition is Most beneficial after liver transplantation for hepatocellular carcinoma in patients with active tumors. Ann Surg. 2020;272(5):855–862. 32889867 10.1097/SLA.0000000000004280

[24] Jeng L , Lee SG , Soin AS , Lee W , Suh K , Joo DJ , et al. Efficacy and safety of everolimus with reduced tacrolimus in living‐donor liver transplant recipients: 12‐month results of a randomized multicenter study. Am J Transplant. 2018;18(6):1435–1446. 29237235 10.1111/ajt.14623

[25] Hua H , Kong Q , Zhang H , Wang J , Luo T , Jiang Y . Targeting mTOR for cancer therapy. J Hematol Oncol. 2019;12(1):71. 31277692 10.1186/s13045-019-0754-1 PMC6612215

[26] Nölting S , Maurer J , Spöttl G , Prada ETA , Reuther C , Young K , et al. Additive anti‐tumor effects of lovastatin and Everolimus In vitro through simultaneous inhibition of signaling pathways. PloS One. 2015;10(12):e0143830. 26636335 10.1371/journal.pone.0143830 PMC4670204

[27] Kim JO , Kim KH , Song IS , Cheon KS , Kim OH , Lee SC , et al. Potentiation of the anticancer effects of everolimus using a dual mTORC1/2 inhibitor in hepatocellular carcinoma cells. Oncotarget. 2016;8(2):2936–2948. 10.18632/oncotarget.13808 PMC5356853 27935857

[28] Zhou H , Fang J , Shang L , Zhang Z , Sang Y , Xu L , et al. MicroRNAs miR‐125b and miR‐100 suppress metastasis of hepatocellular carcinoma by disrupting the formation of vessels that encapsulate tumour clusters. J Pathol. 2016;240(4):450–460. 27577856 10.1002/path.4804

[29] Finn RS , Qin S , Ikeda M , Galle PR , Ducreux M , Kim TY , et al. Atezolizumab plus bevacizumab in unresectable hepatocellular carcinoma. New Engl J Med. 2020;382(20):1894–1905. 32402160 10.1056/NEJMoa1915745

[30] Murakami N , Mulvaney P , Danesh M , Abudayyeh A , Diab A , Abdel‐Wahab N , et al. A multi‐center study on safety and efficacy of immune checkpoint inhibitors in cancer patients with kidney transplant. Kidney Int. 2021;100(1):196–205. 33359528 10.1016/j.kint.2020.12.015 PMC8222056

