# Supplementary material for: Clinical significance of mechanistic target of rapamycin expression in vessels that encapsulate tumor cluster‐positive hepatocellular carcinoma patients who have undergone living donor liver transplantation
Source: Ann Gastroenterol Surg. 2023 Aug 28;8(1):163–71. doi: 10.1002/ags3.12735 (PMC10797838; doi:10.1002/ags3.12735)
Supplement: Supplementary file 1 — Appendix S1. [file AGS3-8-163-s001.pptx]

## Slide 1
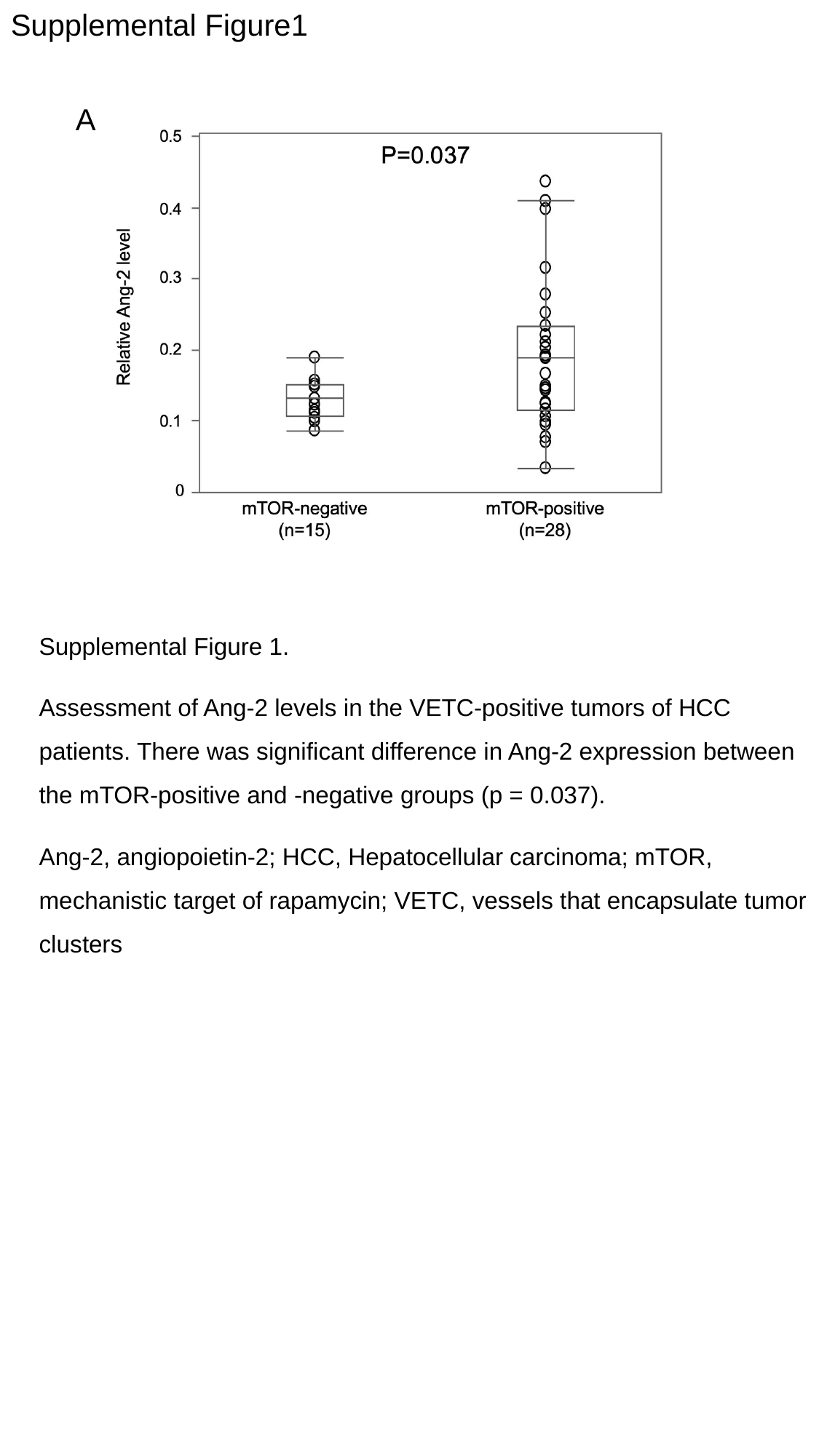

Supplemental Figure1
A
Supplemental Figure 1.
Assessment of Ang-2 levels in the VETC-positive tumors of HCC patients. There was significant difference in Ang-2 expression between the mTOR-positive and -negative groups (p = 0.037).
Ang-2, angiopoietin-2; HCC, Hepatocellular carcinoma; mTOR, mechanistic target of rapamycin; VETC, vessels that encapsulate tumor clusters

## Slide 2
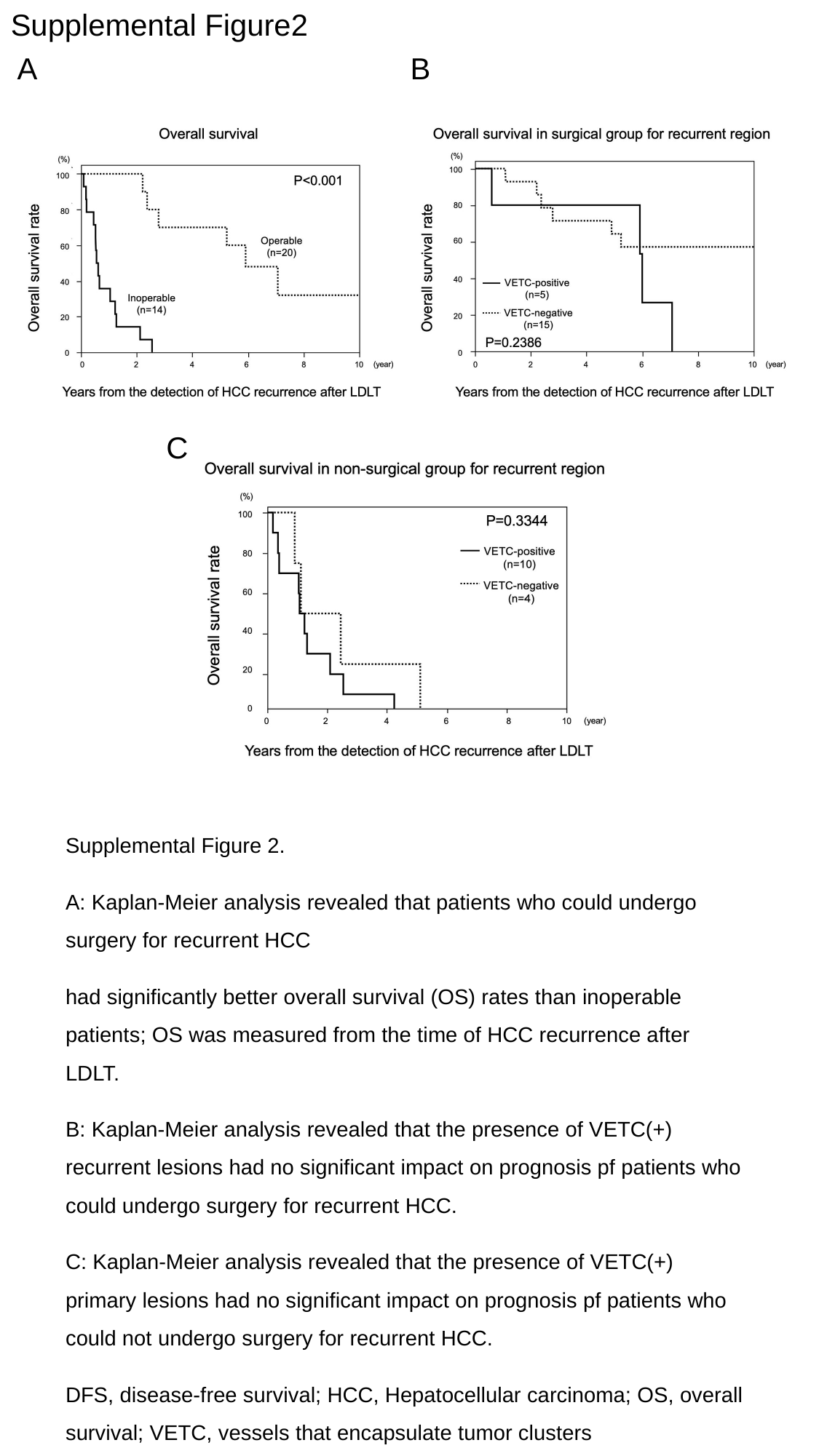

Supplemental Figure2
A
B
C
Supplemental Figure 2.
A: Kaplan-Meier analysis revealed that patients who could undergo surgery for recurrent HCC
had significantly better overall survival (OS) rates than inoperable patients; OS was measured from the time of HCC recurrence after LDLT.
B: Kaplan-Meier analysis revealed that the presence of VETC(+) recurrent lesions had no significant impact on prognosis pf patients who could undergo surgery for recurrent HCC.
C: Kaplan-Meier analysis revealed that the presence of VETC(+) primary lesions had no significant impact on prognosis pf patients who could not undergo surgery for recurrent HCC.
DFS, disease-free survival; HCC, Hepatocellular carcinoma; OS, overall survival; VETC, vessels that encapsulate tumor clusters
